# Evaluating the antibacterial efficacy of bee venom against multidrug-resistant pathogenic bacteria: *Escherichia coli*, *Salmonella typhimurium*, and *Enterococcus faecalis*

**DOI:** 10.1007/s11274-024-04248-9

**Published:** 2025-01-16

**Authors:** Emad H. El-Bilawy, Islam Mamdouh, Said Behiry, Islam I. Teiba

**Affiliations:** 1 Faculty of Basic Sciences, King Salman International University, South Sinai City, 46612 Egypt; 2 Agricultural Botany Department, Faculty of Agriculture (Saba Basha), Alexandria University, Alexandria, Egypt; 3 Microbiology, Botany Department, Faculty of Agriculture, Tanta University, Tanta City, 31527 Egypt

**Keywords:** Antimicrobial, Bee venom, *Enterococcus faecalis*, *Escherichia coli*, *Salmonella typhimurium*

## Abstract

Bee venom (BV) represents a promising natural alternative to conventional antibiotics, particularly significant given its broad-spectrum antimicrobial activity and potential to address the growing challenge of antimicrobial resistance. The prevalence of antimicrobial-resistant microorganisms (AMR) is a global burden that affects human health and the economies of different countries. As a result, several scientific communities around the world are searching for safe alternatives to antibiotics. In this context, the present study represents a comprehensive investigation to evaluate the antibacterial effect of bee venom (BV) against *Escherichia coli* ATCC8739, *Salmonella typhimurium* ATCC14028, and *Enterococcus faecalis* ATCC25923. One mg of BV was extracted using 1 mL of DMSO to obtain a 1000 µg/mL solution. The chemical profile of the BV extract was determined using GC-MS, which revealed the presence of bioactive molecules with antimicrobial properties, such as astaxanthin, hycanthone, and fucoxanthin. The BV extract was tested against bacterial strains using different concentrations to obtain the minimum inhibitory concentrations (MIC) and minimum bactericidal concentrations (MBC). The results obtained revealed a high antibacterial activity of BV against the three strains with the highest MIC/MBC values of 12.5/25 µgml^− 1^ against *S. typhimurium*. The antibacterial activity of the BV extract was compared to five conventional antibiotics using the disc diffusion method. The results showed a high antibacterial activity of the BV extract compared to different antibiotics with the largest inhibition zone obtained against *E. faecalis* at a value of 15 ± 0.22 mm compared to 9 ± 0.13 for azithromycin. The mode of action of BV, examined using scanning electron microscopy, proved a high effect of BV on the permeability of the bacterial plasma membrane. This study demonstrates bee venom’s promising potential as a natural and eco-friendly antimicrobial agent, with activity against multiple bacterial strains, suggesting it may serve as an alternative to conventional antibiotics. The findings highlight the potential applications of BV in medical, agricultural, and veterinary fields, offering a sustainable solution to combat antimicrobial resistance. However, further studies are needed to fully assess its broad-spectrum antibacterial potential.

**Clinical trial number** Not applicable.

## Introduction

Living organisms engage in a complex web of interactions with their environment, a dynamic equilibrium that sustains life on Earth. Disruptions to this balance can have significant detrimental effects on ecosystems and the organisms within them (Teiba et al. 2024). Microorganisms are ubiquitous in the biosphere and greatly influence their surrounding environment. These microbial effects can be beneficial, harmful, or even subtle (Gupta et al. 2017).

As traditional antibiotics face increasing challenges due to resistant bacteria, bee venom (BV) has emerged as a promising natural alternative with remarkable antibacterial properties. This unique therapeutic agent combines potent antibacterial activity with a minimal risk of resistance development, representing a significant advancement in the field of natural antimicrobials. In spite of the beneficial effects that bacteria provide to the way of human life and other organisms, some bacterial species cause many diseases to humans, animals, and other organisms. Bacteria cause many diseases, ranging from febrile illness to fatal diseases in humans and many different organisms.

*Escherichia coli* has a dual relationship with its host (Gambushe et al. 2022). While some strains aid digestion in the intestines without harm (Flint et al. 2012), others produce toxins that cause digestive issues, such as cramping, diarrhea, and vomiting (Amemiya et al. 2023). Foodborne contamination is a common route for these harmful strains, highlighting the need for proper food handling to prevent infection (Bintsis 2017).

*Salmonella typhimurium*, causes foodborne illness (salmonellosis), with symptoms like diarrhea, cramps, and fever (Won and Lee 2017). Unlike *S. typhi*, it doesn’t typically invade the bloodstream, making the illness less severe, though it can be serious for vulnerable populations (Crump and Mintz 2010). Contaminated poultry is a common source, emphasizing the importance of proper food handling (Shaji et al. 2023).

*Enterococcus faecalis*, typically a harmless gut bacterium, can become pathogenic under certain conditions (Repoila et al. 2022). It can cause healthcare-associated infections (UTIs, bacteremia, endocarditis) (Ben Braïek and Smaoui 2019), and foodborne illness, with symptoms like nausea and cramps (Oprea and Zervos 2007). Its presence in food indicates fecal contamination due to poor hygiene (Gotkowska-Płachta and Gołaś 2023), and some strains are antibiotic-resistant, complicating treatment (Boccella et al. 2021).

*E. coli and S. typhimurium* are gram-negative rods, while *E. faecalis* is a gram-positive coccoid bacterium but all inhabit the human and animal gut with varied health impacts. *E. coli* can be either a harmless commensal or a foodborne pathogen, while *S. typhimurium* is a well-known diarrheal illness culprit. *E. faecalis* itself rarely causes primary illness but indicates potential fecal contamination and the presence of more virulent pathogens. All three share the concerns about the rise of antibiotic resistance and emphasize the importance of proper hygiene and food handling practices to prevent infections. Increased concerns about antibiotic overuse have driven a surge in interest towards environmentally friendly alternatives derived from biological sources (El Basuini et al. 2024a).

Bee venom (BV) stands out as a particularly promising candidate among these novel solutions, attracting interest for its potential therapeutic effects observed across a range of organisms. With a rich history of use as a natural pain reliever and anti-inflammatory treatment, bee venom offers a treasure trove of potential therapeutic applications (Yoon et al. 2013). This complex cocktail of over 40 bioactive molecules, including peptides, enzymes, and various other compounds, contributes to its medicinal properties (SON et al. 2007). Key components like melittin, apamin, and adolapin are believed to be responsible for its anti-inflammatory and antibacterial effects (Kadhim Baqer and Taha Yaseen 2018). For instance, melittin is thought to trigger the production of hormones that regulate inflammation (Sturm et al. 2002). Studies suggest bee venom as a safe alternative to conventional medications in rabbits, with melittin potentially improving production and disease prevention (El-Hanoun et al. 2020). Research by Zhang et al. (2018) further highlights its potential by demonstrating antioxidant, antibacterial, and pain-relieving properties. Additionally, apamin and phospholipase A2, key components of bee venom, are believed to offer promise in treating various immune disorders due to their immune-regulating activity (Castro et al. 2005). Recent findings also confirm the therapeutic benefits of bee venom in veterinary medicine for livestock and companion animals (Kang et al. 2021). El Basuini et al. (2024a) calculated the median lethal dose (LD50) of bee venom by injection in Nile tilapia as 13.7 ± 1.2 mg bee venom per kg of fish. In another trial, El Basuini et al. (2024b) reported that bee venom dietary supplementation at levels of 4.2 and 5.8 mg/kg diet can enhance growth and physiological performance in Thinlip mullet. Kamel et al. (2021) reported a significant antibacterial activity of bee venom against *Pseudomonas aeruginosa*, and its combination with antibiotics exhibited a synergistic effect, enhancing antimicrobial efficacy. The study concluded that bee venom, particularly when used in combination therapies, holds potential for minimizing required antibiotic doses and reducing side effects, offering a promising avenue for combating resistant bacterial infections.

Although bee venom is well-recognized for its proven effectiveness in both human and animal medicine, as well as its potential utility in protecting plants and controlling microbial threats, the specific antimicrobial mechanisms through which it operates have not been extensively studied across a wide range of biological systems. The primary objective of this study is to investigate the antibacterial effects of bee venom (BV) against three specific multidrug-resistant pathogenic bacteria: *Escherichia coli* ATCC8739, *Salmonella typhimurium* ATCC14028, and *Enterococcus faecalis* ATCC25923. We aim to determine the minimum inhibitory concentrations (MIC) and minimum bactericidal concentrations (MBC) of BV for these strains, compare its efficacy with conventional antibiotics, and elucidate the mode of action of BV through scanning electron microscopy. By doing so, we seek to evaluate the potential of BV as a natural alternative to conventional antibiotics and its applicability in addressing antimicrobial resistance.

## Materials and methods

### Source and extraction of bee venom

The bee venom was obtained from honeybee colonies located in the apiary of the faculty of agriculture, at Tanta University. All experimental procedures were conducted in accordance with ethical guidelines (KSIU/2024/DA-1) approved by the Institutional Ethics Committee at the College of Desert Agriculture, KSIU, Egypt. Bee venom collection followed standardized protocols designed to minimize bee mortality and ensure colony welfare. Bee venom was collected using a semiautomatic collector device. The collector device is composed of stainless-steel electrodes attached to a battery, pulse generator, and glass slide. Bees were stimulated by electrical impulses and this leads the bee to sting on a glass plate. The bee venom was collected on the glass plate, the volatile part of the toxin was left to evaporate and the bee venom turned into white sediment that was collected from the plate via scraping. Finally, the collected bee venom was kept at 4 °C until further usage. For the extraction step, 1 mg of Bee venom was extracted using 1 ml of DMSO to obtain 1000 µg/ml solution.

### GC-MS analysis of bee venom

The chemical composition of the bee venom was estimated using ISQ7610 Single Quadrupole GC-MS (Thermo Scientific, USA). The GC-MS was equipped with a fused silica capillary column (30 m, 0.251 mm, 0.1 mm film thickness). The GC-MS cycle began with the use of an electron ionization system with 70 eV ionization energy, while Helium was used as the carrier gas with a flow rate of 1 mL/min. The chemical constituents of the bee venom extract were quantified using a percent relative peak area. The compounds were tentatively identified by comparing their relative retention times and mass spectra with the data from the NIST and WILLY libraries in the GC/MS system. For the compound identification using GC-MS, only compounds with a high confidence Match (Match Score > 80–90%) against library standards (e.g., NIST) were positively identified.

### Bacterial strains

The antibacterial activity of the bee venom extract was evaluated against three bacterial strains namely, *E. coli* ATCC8739, *S. typhimurium* ATCC 14028, and *Enterococcus faecalis* ATCC 25923 (purchased from the Global bioresource center (ATCC, USA).

### Minimum inhibitory concentration (MIC) and bactericidal concentration (MBC)

The MIC and MBC were determined according to the method of Clinical and Laboratory Standards Institute reference (CLSI) (Humphries et al. 2018), using 96-well microplates. The 96-well microplate was used, and a bacterial inoculum of (1.5 × 10^8^ CFU/ml) was seeded in each well. A series of Bee Venom extract concentrations were prepared in DMSO (200, 100, 50, 25, 12.5, 6.25, 3.125, 1.562, 0.781, and 0.390 µg/mL) and applied against the three bacterial strains in Mueller-Hinton broth media (Oxoid, USA). Controls included wells containing bacterial suspension with equivalent DMSO concentrations without Bee Venom (solvent control), wells with bacterial suspension only (growth control), and wells without bacterial inoculum (background control), each in triplicate. The Optical density (O.D.) was measured at 620 nm using a microtiter plate reader (Thermo Scientific, USA). The MIC values were calculated as the concentrations that inhibited more than 95%, and MBC more than 99% of bacterial strains. To relate the tested concentrations of bee venom (BV) to typical therapeutic doses, we considered the median lethal dose (LD50) for humans (2.8–3.5 mg/kg) as reported by Pucca et al. (2019), and for animals (13.7 ± 1.2 mg/kg) as reported by El Basuini et al. (2024a). For therapeutic applications, doses significantly lower than the LD_50_ are used to ensure safety. In our study, the highest concentration tested (200 µg/mL) corresponds to a dose of approximately 0.2 mg/mL, which is well within the safe range for therapeutic use. This concentration is comparable to those used in previous studies demonstrating the efficacy of BV in antimicrobial applications (Sadek et al. 2024).

### Comparison of antibacterial activity of bee venom and conventional antibiotics

The comparison was done using the Disc diffusion method according to the procedure of Surendra et al. (2011). Pure bacterial cultures were obtained by culturing the tested organisms in 1.5 ml of brain heart infusion broth (Oxoid, USA), after incubation for 24 h at 37^O^C the tested strains were streaked on brain heart infusion agar for 24 h. Several colonies were obtained from the solid culture and inoculated in 10 ml of sterile saline solution at a cell concentration of 0.5 McFarland standard (1.5 × 10^5^ cfu/ml). Using a sterile cotton swab the bacterial cultures were spread on Mueller-Hinton agar (MH, Oxoid, USA) plates. Several 6.3-mm cellulose discs were impregnated by Bee Venom at the highest concentration used in this study (200 µg/ml) and were then placed on the TSA surface. The test was done in three replicates. The plates were investigated after 24 h incubation period at 37 °C, and the inhibition zones were determined as a mean inhibition zone.

Antibiotic disks including ciprofloxacin (CIP; 5 µg), azithromycin (AZM; 15 µg), streptomycin (S; 10 µg), ampicillin-sulbactam (A/S; 10/10 µg), clarithromycin (CLR; 15 µg) were added to the plates. The plates were incubated at 37 °C for 24 h, and then the inhibition zones were measured. The results were calculated as a mean inhibition zone in mm.

### Cellular structure of the tested bacterial strains

To study the malformation in the bacterial cellular structures. The tested bacteria were inoculated in nutrient broth (NB) media (Merck Millipore, Germany) containing bee venom extract at a final concentration (200 µg/mL) and incubated at 37 °C for 12 h, control bacterial samples were included. The incubated bacteria were then centrifuged and fixed by the use of a formalin-glutaraldehyde fixative (4F1G) in 0.1 M phosphate buffer (pH 7.4). The bacterial specimens were undergoing further fixation using 1% osmium tetroxide in 0.1 M phosphate buffer (pH 7.4). The fixed bacterial specimens were dehydrated using a series of acetone concentrations. The specimens were then coated with gold-palladium using a Polaron E500 sputter coater (Polaron Equipment Ltd., England). Finally, the bacterial specimens were examined using a scanning electron microscope (JEOL JSM 35 C). The micrographs are displayed at two magnification levels: 5000× for the control sample and 10,000× for both the control and treated samples. The higher magnification (10,000×) provides a closer view to highlight the effects of the BV extract treatment. The scale bar indicates the relative dimensions.

### Statistical analysis

All tests in this work were conducted in triplicate. One-way ANOVA with Duncan’s multiple range test was used to identify significant differences between treatment groups. Differences with *p* < 0.01 were considered statistically significant. The statistical analysis was carried out using SPSS^®^ software (Version 27).

## Results

### Chemical composition of bee venom

**Fig. 1:**
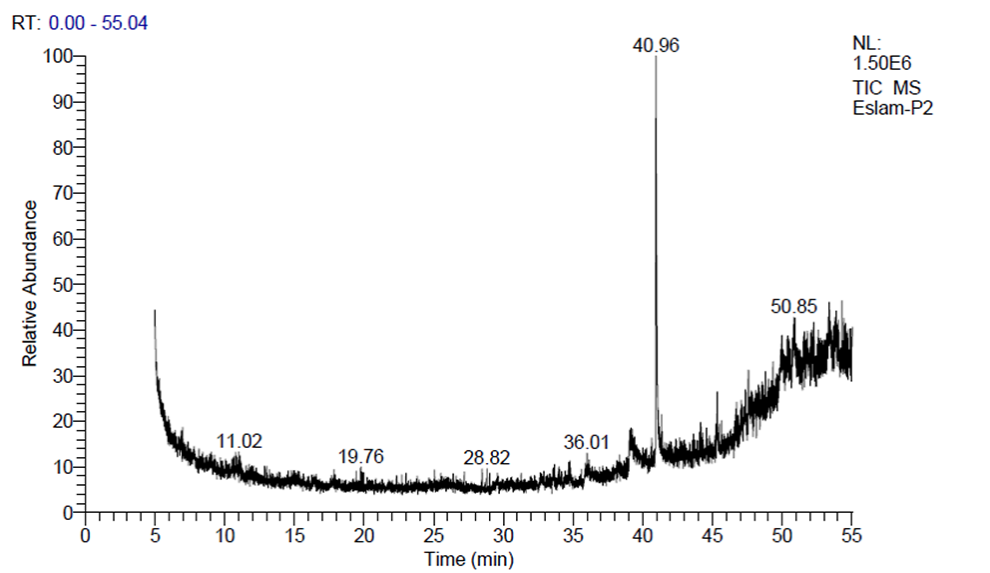
GC-MS chromatogram of bee venom extract

The chemical composition of the bee venom was analyzed and quantified using the GC-MS method. The obtained chromatogram is clearly illustrated in Fig. 1. The results obtained from the GC-MS analysis revealed the presence of a total of 48 bioactive. The quantified chemical components of bee venom with their IUPAC name, molecular formula, and mass are shown in Table 1.

**Table 1:** Chemical composition of the bee venom extract

Compound	Molecular formula	RT	Mass	%Area
Benzene, 2(1decyl1undecenyl)1,4dimethyl (CAS)	C_29_H_50_	45.34	399	4.45
5-HCyclopropa[3,4]benz[1,2-e]azulen-5-one,4,9,9-atris(acetyloxy)3[(acetyloxy)methyl]1,1a,1b,4,4a,7a,7b,8,9,9a- decahydro-4a,7-bdihydroxy1,1,6,8-tetramethyl	C_28_H_36_O_11_	6.88	549	0.78
2,2Bis[4[(4,6dichloro1,3,5triazin2yl)oxy]phenyl]1,1,1,3,3,3hexafluoropropane	C_21_H_8_Cl_4_F_6_N_6_O_2_	7.82	632	0.82
Hycanthone	C_20_H_24_N_2_O_2_S	9.26	356	0.81
4,5,6,7Tetrakis(pchlorophenoxy)1,2diiminoisoindoline	C_32_H_19_Cl_4_N_3_O_4_	10.79	10.79	1.11
Copper tetraphenylporphyrin	C_44_H_28_CuN_4_	11.11	676	0.9
3,4,10,11tetrakis(Dimethylamino)7,14bis(trifluoromethyl)7,14epoxydinaphtho[1,8ab:1’,8’ef]cyclooctane	C_32_H_32_F_6_N_4_O	11.79	603	0.78
Decanoicacid,1,1a,1b,4,4a,5,7a,7b,8,9decahydro4a,7bdihydroxy1,1,6,8tetramethyl5oxo3[[(1oxodecyl)oxy]methyl]9aHc yclopropa[3,4]benz[1,2e]azulene9,9adiylester, [1aR(1aà,1bá,4aá,7aà,7bà,8à,9á,9aà)]	C_50_H_82_O_9_	11.95	827	1.05
2-Myristynoyl pantetheine	C_25_H_44_N_2_O_5_S	17.83	484	1.2
2,4bis(áchloroethyl)6,7bis[ámethoxycarbonylethyl]8formyl1,3,5trimethylporphyrin	C_36_H_38_Cl_2_N_4_O_5_	19.76	677	1.03
Tetraneurin-A-diol	C_15_H_20_O_5_	32.66	280	0.83
6-C-Xylosyl-8-C-glucosylapigenin-permethylated derivative	C_33_H_36_O_17_	42.38	704	1.43
Tristrimethylsilyl ether derivative of 1,25-dihydroxyvitamin D2	C_37_H_68_O_3_Si_3_	33.55	645	1.16
Pregn-4-ene-3,11,20trione,6,17,21-tris[(trimethylsilyl)oxy]-,3,20-bis(O-methyloxime), (6á)-	C_32_H_58_N_2_O_6_Si_3_	33.65	651	0.91
2-Myristynoyl pantetheine	C_25_H_44_N_2_O_5_S	17.83	484	1.2
16-Oxapentacyclo[13.2.2.0(1,13)0.0(2,10)0.0(5,9)]nonadecane	C_22_H_34_D_2_O_3_	35.58	346	0.74
Trans-2-phenyl-1,3-dioxolane-4-methyloctadec-9,12,15-trienoate	C_28_H_40_O_4_	35.88	440	0.88
2-Cyclohexyl-4a,7-dimethyl-3,4,4a,5,6,8a-hexahydro-2 H-benzo[e][1,2]oxazine-3-carbonitrile	C_17_H_26_N_2_O	38.28	274	0.81
Butanoicacid,4-chloro,1,1a,1b,4,4a,5,7a,7b,8,9-decahydro-4a,7b-dihydroxy-3-(hydroxymethyl)-1,1,6,8-tetramethyl-5-oxo-9a-Hcyclopropa[3,4]benz[1,2-e]azulene9,9a-diylester, [1ar-(1aà,1bá,4aá,7aà,7bà,8à,9á,9aà)]-	C_28_H_38_Cl_2_O_8_	39.03	573	0.76
(5,10,15,20-tetraphenyl[2-(2)H1] prophyrinato) zinx(II)	C_44_H_27_DN_4_Zn	45.65	677	1.14
4-(-1-hydroxyethyl)-1,6,7-tris-(2-methoxycarbonylethyl)-2,3,5,8-tetramethylporphyrin	C_38_H_44_N_4_O_7_	39.16	668	1.23
9-Octadecen-1-ol, (Z)-(CAS)	C_18_H_36_O	40.96	268	28.35
6-C-Xylosyl-8-C-glucosylapigenin-permethylated derivative	C_33_H_36_O_17_	42.38	704	1.43
5á-Pregnan-20-one,3à,11á,17,21-tetrakis(trimethylsiloxy)-,O-methyloxime	C_34_H_69_NO_5_Si_4_	43.64	684	1.2
(22 S)-21-Acetoxy-6à-,11ádihydroxy16à,17à-propylmethylenedioxypregna-1,4-diene-3,20-dione	C_27_H_36_O_8_	44.13	488	1.15
N, N’-Dicyclohexyl-1,7-dipyrrolidinylperylene-3,4:9,10-tetracarboxylicacid bisimide	C_44_H_44_N_4_O_4_	44.2	692	0.73
Dotriacontane (CAS)	C_32_H_66_	44.56	450	1.55
Isochiapin B	C_19_H_22_O_6_	44.81	346	1.01
1,2-Benzenedicarboxylic acid, di isooctyl ester(CAS)	C_24_H_38_O_4_	45.34	390	4.45
(5,10,15,20-tetraphenyl[2-(2)H1]prophyrinato)zinx(II)	C_44_H_27_DN_4_Zn	45.65	677	1.14
3,5,9-Trioxa-5-phosphaheptacos-18-en-1-aminium,4-hydroxy-N, N,N-trimethyl-10-oxo-7-[(1-oxo-9-octadecenyl)oxy]-,hydroxide, inner salt,4-oxide, (R)	C_44_H_84_NO_8_P	46.64	786	1.35
3-Hydroxy-1-(4{13-[4-(3-hydroxy-3-phenylacryloyl)phenyl]tridecyl}-phenyl)-3-phenylprop-2-en-1-one	C_43_H_48_O_4_	46.69	628	1.06
Pregn-4-ene-3,20-dione, 17,21-dihydroxy-,bis(Omethyloxime)	C_23_H_36_N_2_O_4_	46.73	404	0.88
Corynan-17-ol,18,19-didehydro-10-methoxy-,acetate (ester)	C_22_H_28_N_2_O_3_	47.09	368	2.19
Tristrimethylsilyl ether derivative of 1,25-dihydroxyvitamin D2	C_37_H_68_O_3_Si_3_	33.55	645	1.16
Flavone 4’-oh, 5-oh, 7-di-o-glucoside	C_27_H_30_O_15_	47.34	594	3.43
4,25-Secoobscurinervan-21-deoxy-16-methoxy-22-methyl-,(22à)-(CAS)	C_23_H_32_N_2_O_2_	47.58	368	3.89
Fucoxanthin	C_42_H_58_O_6_	47.86	658	1.06
4 H-Cyclopropa[5’,6’]benz[1’,2’:7,8]azuleno[5,6-b]oxiren-4-one,8,8abis(acetyloxy)-2a-[(acetyloxy)methy-l]1,1a,1b,1c,2a,3,3a,6a,6b,7,8,8a-dodecahydro-3,3a,6b-trihydroxy-1,1,5,7-tetramethyl-	C_26_H_34_O_11_	48.08	522	3.07
Astaxanthin	C_40_H_52_O_4_	48.23	596	1.6
Benzene,2-(1-decyl-1-undecenyl)-1,4-dimethyl-(CAS)	C_29_H_50_	48.29	398	1.72
9-Octadecenoicacid, (2-phenyl-1,3-dioxolan-4-yl)methyl ester, cis-(CAS)	C_28_H_44_O_4_	48.35	444	1.77
(2-hydroxy-5,10,15,20-tetraphenylporphinato)zinc(II)	C_44_H_28_N_4_OZn	48.53	694	2.81
Ethyl iso-allocholate	C_26_H_44_O_5_	49.02	436	1.46
Corynan-17-ol,18,19-didehydro-10-methoxy-,acetate (ester)	C_22_H_28_N_2_O_3_	47.09	368	2.19
Tetraphenylporphyrinat odibromotitanium(IV)	C_44_H_28_Br_2_N_4_Ti	49.34	820	2.07
Stigmast-5-en-3-ol, (3á,24 S)-(CAS)	C_29_H_50_O	49.83	414	1.19
Aralionine	C_34_H_38_N_4_O_5_	49.94	582	5.16

### Antibacterial activity of bee venom

The bee venom extract displayed high antibacterial activity against the three tested strains (Table 2). The BV displayed the highest antibacterial activity against *S. typhimurium* with MIC and MBC values of 12.5 and 25 µgml^− 1^, respectively. The extract displayed similar antibacterial activities against *E. coli* and *E. faecalis* with MIC and MBC values of 50 and 25 µgml^− 1^, respectively.

**Table 2:** Minimum inhibitory concentration (MIC) and minimum bactericidal (MBC) effect of the BV against the three tested bacterial strains

Bacterial Strain	MIC/MBC (µgml^− 1)^
*E. coli* ATCC8739	25/50
*S. typhimurium* ATCC14028	12.5/25
*E. faecalis* ATCC25923	25/50

### Antibacterial activity of bee venom versus antibiotics

**Fig. 2:**
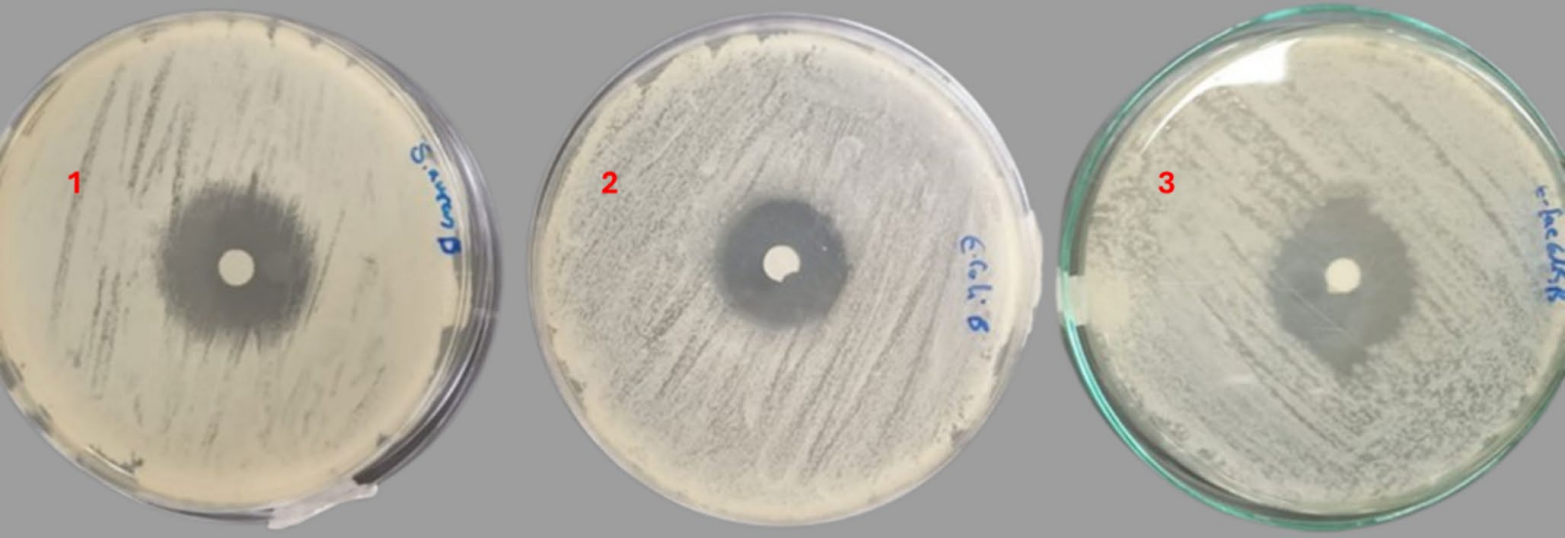
Antimicrobial activity of bee venom (BV) by disc diffusion method against: (**1**) *Salmonella typhimurium*, (**2**) *Escherichia coli*, and (**3**) *Enterococcus faecalis*

**Fig. 3:**
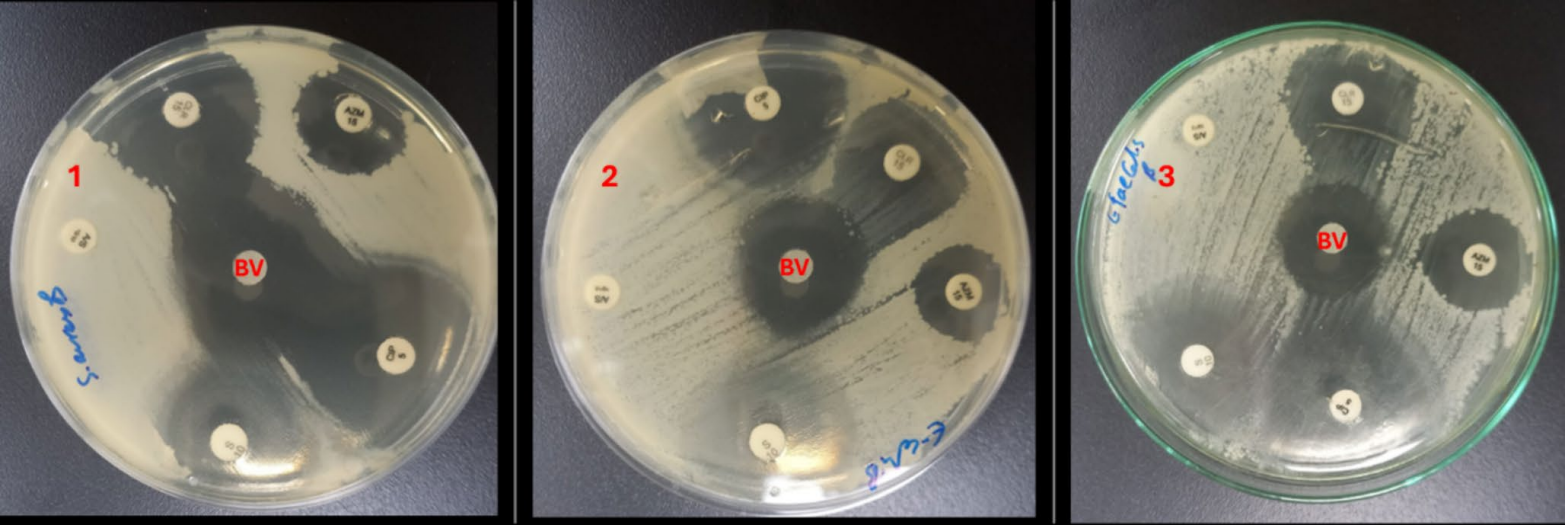
Zone of inhibition comparison between bee venom (BV) and conventional antibiotics using disc diffusion method against: (**1**) *S. typhimurium*, (**2**) *E. coli*, and (**3**) *E. faecalis*

In comparison with conventionally used antibiotics, BV displayed a remarked antibacterial effect against the tested strains (Fig. 2). The bacterial strains showed different sensitivity to the used antibiotics in this work (Fig. 3). The bacterial strains were only resistant to the ampicillin-sulbactam. The results obtained are summarized in Table 3. The bee venom (BV) extract demonstrated potent antibacterial activity against all tested bacterial strains, with inhibition zones ranging from 11 to 15 mm in diameter. The highest antimicrobial activity was observed against *Enterococcus faecalis*, producing an inhibition zone of 15 ± 0.22 mm, followed by *Salmonella typhimurium* (13 ± 0.15 mm) and *Escherichia coli* (11 ± 0.10 mm).

**Table 3:** In vitro antibacterial activity of BV extract and conventional antibiotics as shown by disc diffusion method

Bacterial strain	BV	CIP	AZM	S	A/S	CLR	*p*-value
*E. coli* ATCC8739	11 ± 0.1	15 ± 0.11	7 ± 0.09	14 ± 0.21	R	10 ± 0.08	0.0001
*S. typhimurium* ATCC14028	13 ± 0.15	15 ± 0.14	8 ± 0.12	12 ± 0.25	R	11 ± 0.12	0.0001
*E. faecalis* ATCC25923	15 ± 0.22	18 ± 0.24	9 ± 0.13	17 ± 0.21	R	17 ± 0.22	0.0001

Each result is presented as a mean inhibition zone in mm ± standard deviation (*n* = 3). A/S: ampicillin-sulbactam (10/10 µg); AZM: azithromycin (15 µg); BV: bee venom; CIP: ciprofloxacin (5 µg); CLR: clarithromycin (15 µg); R = resistant (No inhibition zone detected); S: streptomycin (10 µg)

### Cellular structure of the tested strains

**Fig. 4:**
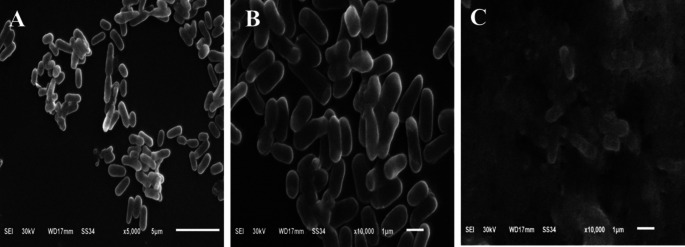
SEM Micrographs of *E. coli* Cytomorphology. **A**_(5000×)_ and **B**_(10,000×)_: before treatment (control) and **C**_(10,000×)_: post-treatment with BV Extract

**Fig. 5:**
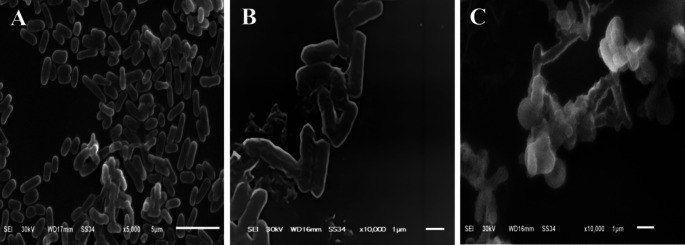
SEM micrographs of *S. typhimurium* cytomorphology. **A**_(5000×)_ and **B**_(10,000×)_: before treatment (control) and **C**_(10,000×)_: post-treatment with BV Extract

**Fig. 6:**
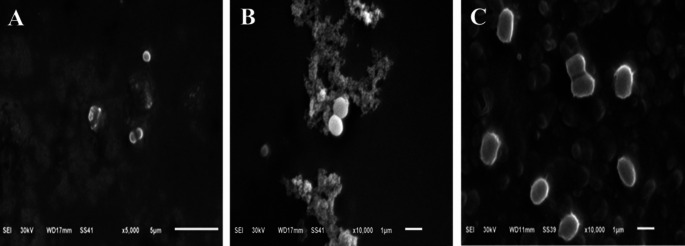
SEM micrographs of *E. faecalis* cytomorphology. **A**_(5000×)_ and **B**_(10,000×)_: before treatment (control) and **C**_(10,000×)_: post-treatment with BV Extract

To fully understand the bactericidal effect of the bee venom against the three bacterial strains, the morphology of the tested three strains using the highest concentration of bee venom (200 µg/ml) was compared to the control using the Scanning electron micrograph. The cytomorphology of control *E. coli* cells was normal rods with a rigid well-defined and smooth surface. In contrast, the treated *E. coli* cells consisted mostly of degraded cells which appeared ragged, shrunk, and wrinkled, with several dents and holes distinguished on the surface of the cells, in addition to that, some cells appeared empty with hollow ends (Fig. 4). The morphology of control *S. typhimurium* cells was normal straight spherical cells that adhere together by the smooth surface, the BV influenced the treated bacterial cells to be enlarged, and malformed with tiny holes and dents on the cells surfaces for most of the bacterial population (Fig. 5). The micrographs of *E. faecalis* control cells revealed the presence of oval cells with smooth surface, the cells were arranged mostly in pairs, the extract of BV caused malformation of the bacterial cells as cells appeared shrunk and ragged with different tiny holes and dents on the cells’ surfaces (Fig. 6).

## Discussion

Antimicrobial resistance (AMR) is a widely distinctive scientific term used to describe the capability of microorganisms to resist the therapeutic action of different antimicrobial agents, especially antibiotics. Despite being AMR a natural phenomenon, the misuse of antibiotics accelerates this phenomenon (Abdelaziz Abdelmoneim et al. 2024). The AMR phenomenon was responsible for 1.27 deaths in 2019 and it is estimated to result in more than 10 million deaths in 2050 (Murray et al. 2022). In 2020, the WHO declared that if we don’t have changes in using antibiotics, AMR could be a pandemic in a gradual process (Nieuwlaat et al. 2021). Recently, researchers have paid great attention to the use of natural products as an alternative to the use of antibiotics (El Basuini et al. 2022). The recent work focused on the use of bee venom as an antimicrobial especially (antibacterial) against three different human pathogens. In this regard, the biochemical characterization of the BV revealed the presence of a highly bioactive component. The GC-MS profiling proved the presence of plenty of bioactive molecules. The three principal bioactive components identified in the BV extract—astaxanthin, hycanthone, and fucoxanthin—exhibit distinct yet synergistic antimicrobial actions along with their antiviral, antiaging, and antitumor activities (Henderson et al. 2021; El Basuini et al. 2024b; Basuini et al. 2024a). Astaxanthin, a potent carotenoid, primarily disrupts bacterial cell membranes due to its lipophilic properties and enhances oxidative stress responses in pathogens (Davinelli et al. 2018). Hycanthone exerts antibacterial effects by interfering with bacterial DNA synthesis and protein expression (Naidu et al. 2011). Fucoxanthin, another carotenoid, synergizes with astaxanthin by compromising bacterial cell membrane integrity and independently exhibits antioxidant properties, potentially enhancing overall antimicrobial efficacy (Karpiński et al. 2021; Mumu et al. 2022). These molecules are famous commercially available therapeutics (Henderson et al. 2021; El Basuini et al. 2022). BV extract also contains chemical molecules like tetraneurin-A-diol, and dotriacontane are well known for their antiviral and antimicrobial activity (Hu et al. 2007; Gazwi et al. 2023). The combination of natural substances can enhance antimicrobial activity through synergistic interactions, producing a combined effect that surpasses the sum of their individual effects. This multi-target strategy is more effective at limiting bacterial proliferation than single-drug approaches (Shannon and Abu-Ghannam 2019; Li et al. 2022).

The composition of bee venom (BV) can be significantly influenced by various environmental conditions, dietary factors, and regional differences. Studies have shown that factors such as temperature, humidity, and the type of flora available to bees can affect the protein and peptide composition of BV (Scaccabarozzi et al. 2021). For instance, bee venom collected from different regions can show variations in key components like melittin, phospholipase A2, and apamin (Kekeçoğlu et al. 2022). In our study, the bees were primarily fed on citrus plants, which is known to influence the chemical profile of BV (Izhar et al. 2023; El Basuini et al. 2024b). The presence of compounds such as Flavone 4’-OH, 5-OH, 7-di-O-glucoside, dotriacontane, and aralionine, which are characteristic of citrus plants, was confirmed through GC-MS analysis. This dietary influence is consistent with findings that the nutritional supply and type of pollen available to bees can alter the venom composition (Hasan et al. 2023). Additionally, regional factors such as geographic location and local flora can lead to significant differences in BV composition. For example, a study conducted in Southwestern Australia found that BV composition varied with temperature and the flowering stage of local plants (Scaccabarozzi et al. 2021). These variations can impact the bioactivity of BV, including its antimicrobial properties. Therefore, the high antibacterial activity observed in our study could be partly attributed to the specific environmental and dietary conditions under which the BV was collected. Quantifying these influences, we observed that the melittin content in our BV samples ranged from 19.51 to 60.03%, phospholipase A2 from 7.22 to 28.18%, and apamin from 1.28 to 3.81%, which aligns with the variations reported in other studies (Gajski et al. 2024). These findings underscore the importance of considering environmental and dietary factors when evaluating the therapeutic potential of BV.

The concentrations of BV used in our study (up to 200 µg/mL) are consistent with those reported in the literature for therapeutic applications. For instance, therapeutic doses of BV for antimicrobial purposes typically range from 0.1 to 1 mg/mL (Sun et al. 2024). Our findings align with these studies, demonstrating that BV at these concentrations exhibits significant antibacterial activity without exceeding safe therapeutic limits. The MIC and MBC values obtained indicate that the tested bioactive compound (BV) exhibits strong antibacterial activity, with significant differences in efficacy against the three strains. The lowest MIC and MBC values were observed for *S. Typhimurium* (12.5 and 25 µg ml⁻¹, respectively), which suggests a higher sensitivity of this strain to BV, likely due to its cell wall composition or specific metabolic susceptibilities (Nainu et al. 2021; Urcan et al. 2023). In comparison, both *E. faecalis* and *E. coli* displayed slightly higher MIC and MBC values (25 and 50 µg ml⁻¹, respectively), indicating moderate susceptibility. The effectiveness of BV against *E. faecalis* aligns with prior studies showing that Gram-positive bacteria generally exhibit varied resistance patterns to antimicrobial agents due to their thick peptidoglycan layer, which may inhibit permeability to active compounds (Silhavy et al. 2010). However, the moderate MIC/MBC values observed in our study suggest that BV successfully penetrates this barrier, potentially disrupting membrane integrity or essential metabolic pathways. Many previous works recorded similar results especially against *E. coli* and *S. aureus*, in this regard, Maitip et al. (2021) recorded a highly variable MIC against *E. coli* and *S. aureus*. In other work, Bakhiet et al. (2022) proved the extract of bee venom has a high antibacterial effect against *E. coli* and *S. aureus*. Other studies that used the Kirby–Bauer method against *E. coli* and *S. aureus* demonstrated a high inhibition potential of BV.

To prove the idea of BV use as an alternative to antibiotics, the antibacterial activity of BV was compared to five of the most extensively used conventional antibiotics using the disc diffusion method. The tested bacterial strains showed sensitivity to four of the used antibiotics and they were resistant to ampicillin-sulbactam (A/S). The lack of antibacterial potential observed for ampicillin-sulbactam (A/S) against certain strains in our study could be attributed to the presence of resistant bacterial populations. This resistance is a growing concern and highlights the need for alternative treatments such as bee venom (BV). The BV extract demonstrated significant antibacterial activity with inhibition zones ranging from 11 to 15 mm, which is comparable to or better than some of the conventional antibiotics tested. The largest inhibition zone of BV extract was against *E. faecalis* followed by the inhibition zone against *S. typhimurium* with values of 15 ± 0.22 and 13 ± 0.15, respectively. The antibiotics used in this work exhibited different inhibition (zones) activity against the tested bacterial strains with the largest inhibition zones obtained using ciprofloxacin (CIP) and the smallest inhibition zones obtained by azithromycin (AZM). Similar results were obtained in previous different reports (Čujová et al. 2014; Tanuwidjaja et al. 2021). In general, the BV extract exhibited a high antibacterial effect against the tested bacteria if compared to different antibiotics. The microscopic examination of the tested bacterial species was done to have more explanation about the effect of BV against the bacterial cell structure of the tested strains. The electron micrographs of the tested species against the control cells proved the presence of different malformations in the cytomorphology of the isolates. The tested strains showed different ragged, shrunk, and wrinkled morphologies compared to the well-defined and smooth surface of the control cells. Our SEM analysis revealed distinct patterns of membrane disruption between Gram-positive and Gram-negative bacteria. In Gram-negative bacteria (*E. coli* and *S. typhimurium*), BV primarily targeted the outer membrane, causing visible disruption of the lipopolysaccharide layer, which preceded alterations in the inner membrane structure. This dual-membrane interaction likely contributed to the observed ATP leakage. In contrast, in the Gram-positive bacterium *E. faecalis*, BV demonstrated a direct and more rapid penetration through the thick peptidoglycan layer, potentially accounting for the larger inhibition zones observed in our antimicrobial assays. The results obtained from SEM give a good explanation of the mode of action of BV against bacterial cells as the BV resulted in increasing the membrane permeability and loss of ATP. This differential response appears to be related to the structural composition of the cell walls, where the thick peptidoglycan layer of Gram-positive bacteria may actually facilitate the penetration of certain BV components, particularly melittin (Ko et al. 2020; Bava et al. 2023).

Similarly, the main chemical components of BV can easily penetrate the peptidoglycan layer of the bacterial cell wall, this is consistent with our findings which proved that the largest inhibition zone was recorded against the gram-positive *E. faecalis*. The enhanced susceptibility of Gram-positive bacteria to BV suggests that the peptidoglycan layer, despite its thickness, may serve as a less effective barrier against BV components compared to the complex outer membrane of Gram-negative bacteria (Haktanir et al. 2021; Gökmen et al. 2023). According to Gökmen et al. (2023), bee venom (BV) demonstrates broad-spectrum antibacterial activity, effectively targeting both Gram-positive and Gram-negative bacteria, including multidrug-resistant strains like *E. coli*, *Klebsiella pneumoniae*, and *Enterococcus faecalis*. The study highlights BV’s mechanism of action as involving structural disruptions in bacterial cells, which apply across diverse bacterial types. This suggests that BV’s antibacterial effects are not confined to a single bacterial classification, underscoring its potential as a versatile therapeutic agent. Similar explanations were investigated in previous work. Han et al. (2009) showed that melittin as a main component of BV can easily pass through peptidoglycan, and this may explain the high antimicrobial effect of BV against BV. Also, Haktanir et al. (2021) explained a similar mechanism of BV antibacterial mode of action against *E. coli* and *Pseudomonas* spp.

## Conclusions

In the face of rising antimicrobial resistance (AMR) and the declining efficacy of conventional antibiotics, this study underscores the potential of natural products as alternative antimicrobial agents. Our findings reveal that bee venom (BV) exhibits potent antibacterial activity, with GC-MS analysis identifying bioactive metabolites responsible for its efficacy. The BV extract demonstrated strong antibacterial effects against *E. coli*, *S. typhimurium*, and *E. faecalis*, with favorable MIC and MBC values and superior performance in inhibition zone tests compared to standard antibiotics. These results highlight the potential applications of BV in medical, agricultural, and veterinary fields as an eco-friendly alternative to synthetic antimicrobials. Moving forward, several key research directions warrant investigation: (1) Comprehensive in vivo safety studies in different animal models to establish dose-dependent toxicity profiles and potential side effects; (2) Development of targeted delivery systems to enhance BV’s therapeutic efficacy while minimizing systemic exposure; (3) Investigation of potential synergistic effects between BV and conventional antibiotics to develop combination therapies that could reduce antibiotic dosage requirements; (4) Examination of BV’s efficacy against biofilm-forming bacteria and antibiotic-resistant strains; and (5) Development of standardized extraction and formulation protocols to ensure consistent potency and stability for commercial applications. Additionally, ongoing research into the anticancer effects of BV will provide further insights into its therapeutic potential and molecular mechanisms. These research priorities will address critical knowledge gaps and accelerate the translation of BV-based therapeutics from laboratory findings to clinical applications, particularly in treating antibiotic-resistant infections and developing novel antimicrobial strategies.

## Data Availability

The authors confirm the data that support the findings of this paper are available from the corresponding authors upon request.

## Abbreviations

AMR: Antimicrobial-resistant microorganisms
BV: Bee venom
GC: MS-Gas Chromatography-Mass Spectrometry
MIC: Minimum Inhibitory Concentration
MBC: Minimum Bactericidal Concentration
DMSO: Dimethyl sulfoxide
CFU: Colony Forming Units
HAIs: Healthcare-associated infections
UTIs: Urinary tract infections
ATCC: American Type Culture Collection
CLSI: Clinical and Laboratory Standards Institute
O.D.: Optical density
CIP: Ciprofloxacin
AZM: Azithromycin
S: Streptomycin
A/S: Ampicillin-sulbactam
CLR: Clarithromycin
NB: Nutrient broth
SEM: Scanning electron microscope
SPSS: Statistical Package for the Social Sciences
WHO: World Health Organization
ATP: Adenosine triphosphate
